# Clinician Perspectives on Digital and Computational Pathology: Clinical Benefits, Concerns, and Willingness to Adopt

**DOI:** 10.3390/diagnostics15192527

**Published:** 2025-10-07

**Authors:** Charu Aggarwal, Aakash Desai, Nicholas McConnell, Nicholas Cadirov, Gary Gustavsen, Arushi Agarwal, Nabil Chehab, Srividya Kotapati, Nikunj Patel

**Affiliations:** 1 Division of Hematology-Oncology, Perelman School of Medicine, Abramson Cancer Center, University of Pennsylvania, Philadelphia, PA 19104, USA; 2 Division of Hematology and Oncology, Department of Medicine, University of Alabama at Birmingham, Birmingham, AL 35294, USA; 3 Health Advances LLC, 275 Grove St. Ste 1-300, Auburndale, MA 02466, USA; 4 AstraZeneca, Wilmington, DE 19803, USA

**Keywords:** digital pathology, computational pathology, survey

## Abstract

**Background/Objectives**: Precision medicine has transformed how we manage cancer patients. As treatments and drug targets become more complex, the associated diagnostic technologies must also evolve to actualize the benefit of these therapeutic innovations. Digital and computational pathology (DP/CP) play a pivotal role in this evolution, offering enhanced analytical techniques and addressing workflow constraints in pathology labs. This study aims to understand clinicians’ awareness, utilization, and willingness to adopt DP/CP-based tools, as well as the role they perceive themselves playing in the adoption of CP-based tests. **Methods**: A double-blinded, online quantitative survey was conducted among 101 U.S.-based medical oncologists. **Results**: Awareness of DP/CP varied among clinicians, with only 17% identifying as very aware. Subsequently, the current utilization of CP-based tests is also low. Despite this, clinicians are optimistic about the potential benefits of DP/CP, including reduced turnaround times, improved therapy selection, and more consistent slide review. To achieve full adoption, clinicians recognize that barriers must be addressed, including cost, regulatory guidance and, to a lesser extent, concerns with the “black box” nature of CP algorithms. While the focus for the adoption of DP has centered on pathologists, clinicians anticipate playing a more significant role in the adoption of CP-based tests. Finally, clinicians demonstrated clear willingness to utilize a CP-based CDx, with 90% of respondents identifying as potential adopters. **Conclusions**: This study highlights a positive outlook for the adoption of DP/CP among clinicians, despite varied awareness and low current utilization. Clinicians recognize the potential benefits of DP/CP but also acknowledge barriers to adoption. Addressing these barriers through education, regulatory approval, and collaboration with pathologists and biopharma is essential for successfully integrating DP/CP technologies into clinical practice.

## 1. Introduction

The emergence of precision medicine has transformed the cancer therapy landscape from generalized, non-specific therapy applied to large patient cohorts to highly refined therapies addressing the needs of specific patients at discrete phases of their journey. Instrumental to this accomplishment was the development of targeted therapies specific to actionable mutations and the lockstep emergence of broadly disseminated testing technologies to identify the correct treatment for each patient. Immunohistochemistry (IHC) is a primary technology in oncology due to its ability to provide vital diagnostic and prognostic information, as well as supporting therapeutic selection for many cancer types [1]. Even with the growth of molecular testing, IHC maintains a strong presence due to its ability to measure a broad range of markers in both a cost- and time-effective manner. However, traditional IHC has shortcomings that are becoming increasingly significant in the rapidly evolving field of oncology testing. The subjectivity of interpretation creates risk for inconsistent results. Furthermore, as therapeutic response biomarkers transition from single markers to complex models and signatures, traditional IHC is insufficient to provide the necessary granularity for accurate interpretation. Finally, IHC is a largely manual process that is becoming increasingly challenging to maintain, given the declining pathologist workforce and increasing overall number of diagnoses [2,3].

Artificial intelligence and machine learning (AI/ML) are enabling the next phase of precision medicine by expanding our methods of interpreting previously unobtainable or impractical data and enabling the use of complex biomarkers that further refine our understanding of patient care [4,5]. One area in which AI/ML is making an impact is within digital and computational pathology (DP/CP). DP/CP is a broad class of technologies comprising hardware and software to digitize slides, archive scanned slides, annotate slide images, and leverage algorithms to expedite and access new capabilities in slide analysis. DP/CP is a rapidly emerging testing technology, offering more objective and enhanced analytical techniques and addressing workflow constraints in the pathology lab [6].

To date, efforts to drive the adoption of DP/CP have emphasized hardware components to alleviate challenges associated with manual anatomic pathology, including an increasing shortage of trained pathologists, the growing complexity of anatomic pathology (AP) tests, inefficiencies with traditional glass slide storage, and the subjectivity of interpreting results [7,8]. While much of the focus within DP/CP has centered on developing the necessary infrastructure through hardware, algorithm- and AI-based tests are increasingly gaining traction, with multiple tests having been approved and in development [9,10,11,12]. Paige Prostate from PaigeAI, approved in 2021, aids in the diagnosis of prostate cancer from H&E-stained slide images of prostate needle biopsies. Roche’s uPATH HER2 Dual ISH has IVD status to aid in determining HER2 status. Digital pathology tools have already demonstrated effective benefits over traditional methods, including virtual staining to automate workflows [13], automating biomarker scoring [14,15], and identifying new biomarkers [16,17].

More recently, quantitative continuous scoring (QCS) of whole-slide images has been used to more effectively stratify HER2 expression and predict the response to trastuzumab deruxtecan [1]. Additionally, QCS was used to identify a novel measure of TROP2 expression based on its localization with tumor cells (known as the normalized membrane ratio, or NMR) and was identified as being capable of predicting the response to datopotamab deruxtecan [18]. These recent studies underscore the growing importance of these sophisticated tools in patient care. As the therapeutic pipeline expands to new mechanisms of action (MOAs), the segmentation of patients by biomarker status is becoming increasingly complex, and traditional testing methods risk missing patients who are likely to respond to treatment. Furthermore, as new and innovative biomarkers are identified—including TROP2 NMR—new methods of segmentation will be required. It will soon be imperative for clinicians to be well-informed and aligned on the use of new technologies for optimizing patient care.

Given DP/CP’s place in the lab and the current emphasis on hardware solutions, pathologists have been heralded as the primary stakeholders, and there have been multiple efforts to understand the pathologist’s perspective to further drive the adoption of DP/CP [19]. Pathologists are likely to remain the key gatekeepers to widespread DP/CP adoption; however, as CP-based tests become further developed, the role of the clinician, as the party responsible for ordering tests and acting on the results to ensure that an appropriate treatment is selected for each patient, will become increasingly important to understand.

To better understand clinicians’ awareness, understanding, and willingness to adopt DP/CP-based tools, we conducted a quantitative survey among medical oncologists across various practice settings, specializations, and self-reported awareness of DP/CP tools.

## 2. Materials and Methods

### 2.1. Survey Methodology

A double-blinded, online quantitative survey was conducted with United States-based medical oncologists (*n* = 101). To ensure variation in respondent demographics, screening questions were implemented to collect responses from a mix of clinicians across multiple categories. The respondent demographics are displayed in Table 1.

The survey sought to evaluate current awareness, perceived benefits, concerns, and willingness to order DP/CP tests. The survey examined both the general application of DP/CP technologies and the willingness to adopt a defined, theoretical FDA-approved DP/CP-based companion diagnostic test using a combination of categorical and Likert-style questions. The survey was designed to accommodate respondents with varying levels of awareness and utilization of DP/CP tools. To ensure consistency of responses, the following definitions of digital and computational pathology were provided at the start of the survey:

***Digital pathology*** *encompasses the acquisition, management, analysis, and interpretation of pathology information generated from digitized glass-slide images.*

***Computational pathology*** *is a subset of digital pathology that uses AI-guided computer-based quantification and classification of tissue morphology to generate more precise diagnoses and expand the recognition of pathologic changes beyond the limits of conventional visual assessment. This could include automatic detection, localization, or quantification of histological parameters and structural changes.*

### 2.2. Interviews

A series of 60 min double-blinded phone-based interviews was performed to inform the development of the quantitative survey with medical oncologists. Questions were asked regarding awareness of computational pathology, as well as drivers and barriers to its use.

### 2.3. Study Participant Selection Criteria

To ensure high-quality research, the 101 survey respondents were recruited by market research vendors in compliance with industry standards. Ethical review and approval were waived for this study by Advarra CIRBI Platform, using the Department of Health and Human Services regulations found at 45 CFR 46.104(d)(2), and the IRB determined that the research project was exempt from IRB oversight.

All survey respondents were required to be board-certified medical oncologists; currently dedicating greater than 25% of their time to direct patient care; and seeing, on average, 5 or more unique patients with lung cancer per month.

## 3. Results

### 3.1. Clinician Awareness, Utilization, and General Comfort with DP/CP

**Figure 1:**
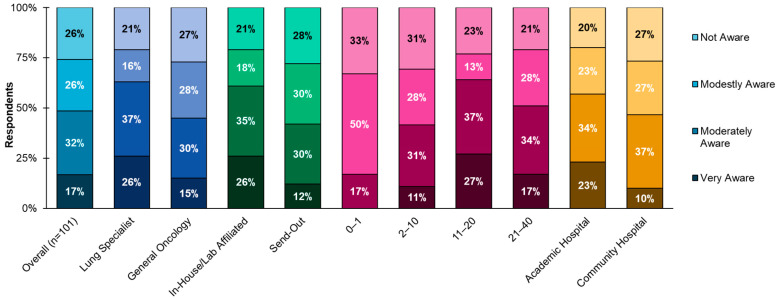
Clinician self-reported awareness of DP/CP. Awareness of DP/CP is highly varied among lung specialists, clinicians with in-house labs, those with more years of experience, and those in an academic setting, with the latter trending toward higher awareness. Lung specialists were defined as respondents who treat 70 or more patients for lung cancer per month. Community hospitals also include those with academic affiliation. Private practice encompasses both independent practices and those affiliated with a network or hospital.

The clinicians’ self-reporting on personal awareness of DP/CP demonstrated a high degree of variability, with only 17% identifying as very aware (knowingly leveraging a digital pathology or computational pathology technology), 25% identifying as having no awareness, and 59% identifying as having modest or moderate awareness of DP/CP (Figure 1). A higher awareness trend was observed among clinicians whose primary practice setting was an academic institution and/or who had an in-house pathology lab. A total of 23% of clinicians from academic institutions reported being “very aware” of DP/CP. Similarly, 26% of clinicians with an in-house pathology lab reported being “very aware” as well. Clinicians practicing for 11–20 years reported the highest awareness, with 27% reporting as being “very aware” compared to 17% of clinicians practicing for 21–40 years and 11% of clinicians practicing for 2–10 years. No clinicians in their first year of practicing reported being very aware of DP/CP.

**Figure 2:**
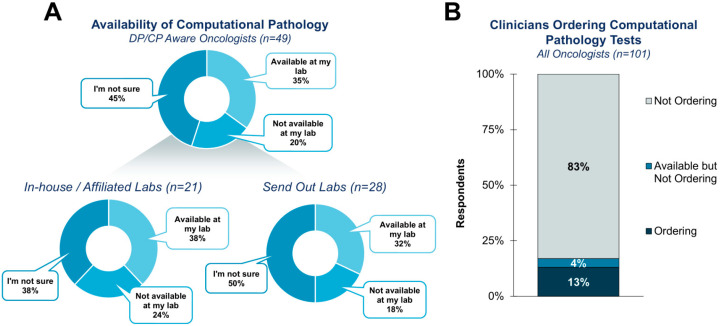
The availability and utilization of computational pathology tests. (**A**) Most clinicians are either unaware of the availability of a computational pathology-based test or knowingly do not have access to one. (**B**) Current utilization is low, as only 13% of current clinicians knowingly order a computational pathology-based test.

Consistent with the varied awareness of DP/CP in general, the current utilization of computational pathology tests is specifically low (Figure 2). Only 13% of respondents reported ordering a computational pathology test. An additional 4% reported having a test available but not ordering it. These results were expected, as most computational pathology utilization is currently hypothesized to be in research and trial settings.

**Figure 3:**
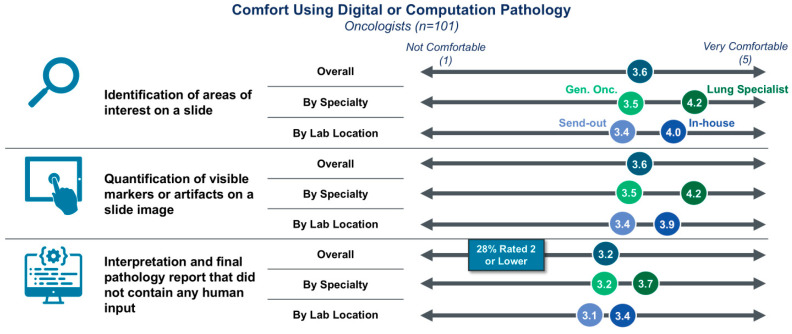
Comfort ordering a DP/CP-based test by use case. Clinicians are generally willing to use results from a DP/CP-based test. Lung specialists and clinicians with an in-house lab are more amenable than their respective counterparts.

Despite the varied awareness and utilization, clinicians are generally amenable to leveraging DP/CP for processing slides (Figure 3). On a scale of 1–5, with 1 being “not comfortable” and 5 being “very comfortable,” clinicians scored concepts of using DP/CP to identify areas of interest on a slide or quantifying visual markers on a slide as a 3.6, with only 12–14% of respondents scoring either use case as a 1 or 2. Greater uncertainty arises when applying DP/CP technologies to the automated interpretation of slides. While the average comfort score for this use case remains above 3 out of 5 at 3.2, 28% of respondents scored the use case as a 1 or 2, a notable minority of clinicians. The increasing concern with this level of testing sophistication underscores the likely need for appropriate education and validation to foster confidence in more advanced testing.

### 3.2. Benefits and Barriers to DP/CP Adoption

**Figure 4:**
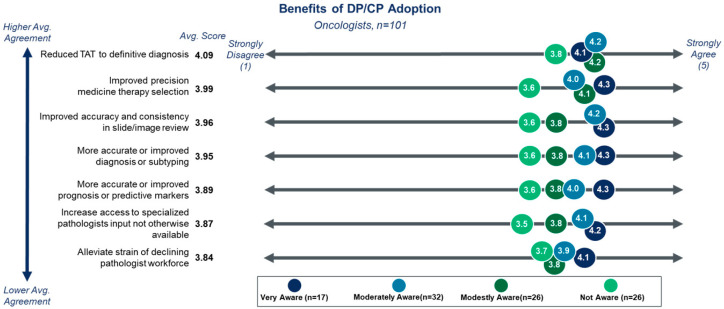
Perceived benefits of adopting DP/CP-based testing. Clinicians are more likely to agree with all proposed benefits of DP/CP than to disagree, with reduced TAT, improved therapy selection, and improved accuracy of slide review being perceived as the most likely benefits. Higher awareness of DP/CP trends is associated with greater agreement on the potential benefits.

Despite the varied awareness and current low utilization, clinicians largely readily agree with the potential benefits of DP/CP technologies (Figure 4). When provided a list of value proposition statements for DP/CP technologies to agree or disagree with on a scale of 1–5, greater than 60% of clinicians agreed with each of the statements. Clinicians most strongly agreed that DP/CP tools could help reduce TAT to a definitive diagnosis, improve therapy selection, and improve consistency in slide review. Each value proposition received 78%, 76%, and 72% agreement, respectively. While agreement, on average, remained high regarding the potential benefits of DP/CP in improving accuracy, diagnosis, or prognosis, the impact on the degree of awareness was more notable for these value propositions. Clinicians with high or moderate awareness had 87% and 84% agreement, respectively, compared to 63% and 60% agreement for those with no awareness. This again highlights a potential focus area for champions of DP/CP to drive awareness and education among colleagues who are less familiar with the topic.

**Figure 5:**
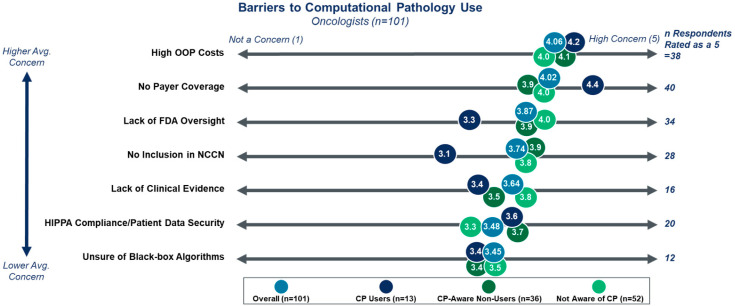
Perceived barriers to DP/CP adoption. Clinicians are moderately to highly concerned with all potential barriers to DP/CP adoption. The highest concern is for barriers common to any novel technology. Current DP/CP users are more likely to be concerned with costs and coverage over regulatory approval. Current users were asked what they perceived as the barriers to CP testing at the time of initial adoption, not the perceived current barriers.

While the overall sentiment regarding DP/CP is positive, clinicians readily acknowledge that numerous barriers exist to the adoption of DP/CP technologies (Figure 5). Clinicians’ largest concerns for DP/CP technologies match traditional systemic barriers for any novel testing technology, as the barriers of the highest concern focus on the cost of testing (high OOP cost and lack of payer coverage) and regulatory guidance (FDA oversight and NCCN guideline inclusion). Current users of CP-based tests had markedly lower concern around FDA clearance and NCCN guideline inclusion than the average response. Given the current state of CP-based tests, it is logical that early adopters would not be deterred by the lack of formal approval. One key question sought to be answered by this survey was the degree of concern surrounding the “black box” concept associated with CP-based testing algorithms. Among those surveyed, concern was limited, resulting in the barrier scoring lowest on average. However, 12% of current non-users expressed “high concern” regarding this concept. As such, efforts to alleviate this concern will be important for DP/CP technologies to reach full adoption.

Consistent with the findings for top barriers, respondents on average identified NCCN guideline inclusion and FDA approval as having the greatest impact on addressing concerns with CP-based tests (Figure S1). Current users were more likely to alter their practice based on peer-reviewed research and endorsements from colleagues.

### 3.3. Clinicians’ Role in DP/CP Adoption

Traditionally, efforts to drive the adoption of DP/CP technologies have focused on pathologists, particularly in adopting digital pathology platforms placed in the pathology lab. Clinicians are largely a minor stakeholder in these conversations. Our survey supported this sentiment, as 45% of clinicians surveyed were uncertain if any DP/CP technologies were currently utilized by their lab (Figure 2).

However, regarding the specific concept of CP-based tests, clinicians appear to have played, or anticipate playing, a larger role in the adoption decision (Figure S2). A total of 55% of clinicians currently using a CP-based test perceive themselves as the primary decision makers in the process. Additionally, nearly 70% of clinicians not currently using the technology anticipate playing a role in the adoption decision of any future CP-based test. This highlights the need to drive education and awareness of CP-based testing among clinicians by expanding the body of evidence through impactful research and effective dissemination, as clinicians are likely to play a key role in the adoption of future tests.

### 3.4. Willingness to Adopt a Theoretical CP-Based CDx Test

As the final part of the survey, we sought to assess clinicians’ willingness to specifically adopt a CP-based CDx test. The following theoretical scenario was presented:

*Assume there is a companion diagnostic for a newly approved targeted therapy in lung cancer. The companion diagnostic test uses computational pathology to precisely quantify the level of a biomarker in tumor cell slide images (i.e., quantifying a human-interpretable feature) to determine positivity and eligibility for the targeted therapy. Assume this is the only test available for this therapy.*

**Figure 6:**
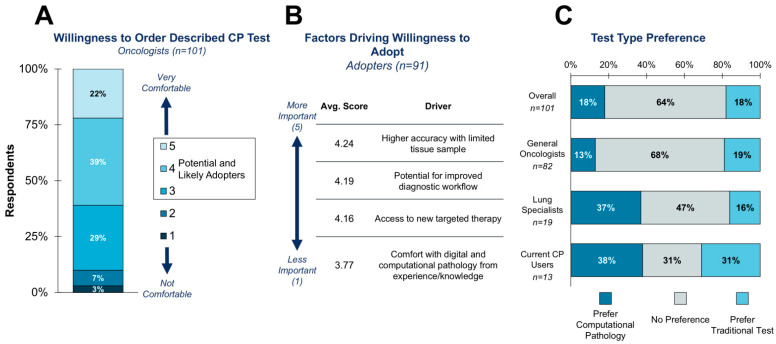
Response to a theoretical CP-based CDx test for a novel lung cancer therapy. (**A**) When asked about their comfort ordering the described test, 90% of clinicians stated they would be at least somewhat comfortable. (**B**) Three drivers for adopting the test scored an average greater than 4: higher accuracy with limited tissue samples, potential for improved diagnostic workflow, and access to a new targeted therapy. (**C**) If given the option for the same test to utilize either a traditional or CP-based test, most clinicians have no preference. Current CP users are split with nearly equal numbers preferring a CP test, having no preference, or preferring a traditional test.

Consistent with earlier findings on the general willingness to adopt DP/CP technologies and the desire for FDA approval for any CP-based test, 90% of clinicians were at a minimum somewhat willing to adopt the test concept provided (Figure 6A). The 10% of those who were unwilling to adopt continues to highlight the small, yet adamant, portion of clinicians with concerns around leveraging CP-based tests.

For those willing to adopt, the primary drivers for adoption were the perceived ability to have more accurate testing, improve diagnostic workflow, and access new targeted therapy, with average scores of 4.24, 4.19, and 4.16, respectively (Figure 6B). Comfort with CP-based testing scored the lowest among the test drivers, with an average score of 3.77. This suggests that clinicians are most driven by the clinical impact of any new test, and technology is a secondary consideration, serving as an important reminder of features to concentrate on for any future CP-based test when seeking to drive clinician support.

When asked about their preference if given the option of a traditional versus CP-based CDx test, most clinicians reported no preference, which is to be expected given the current high variability in awareness (Figure 6C). However, an unexpected finding was that over 30% of current CP users would prefer a traditional pathology test. This finding suggests that challenges with CP-based tests likely exist, which may pose a risk to future tests. Future work will aim to better understand the pain points that clinicians face when ordering CP-based tests in order to improve future technologies in the field.

## 4. Discussion

**Figure 7:**
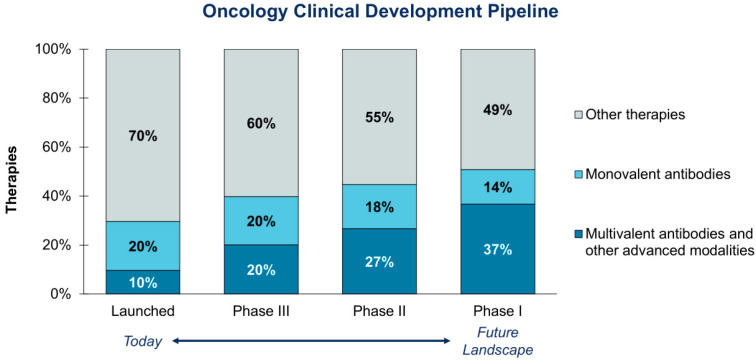
Biopharma oncology pipeline by modality. An increasing share of therapies in development are multivalent antibodies or other advanced modalities that will require new diagnostic technologies, such as DP/CP.

While precision medicine has already provided immense benefit to cancer patients, there is still additional work to be completed. Response rates to targeted therapies often remain low, the benefits can be short-lived, and many patients still have few to no targeted treatment options available. Further, it is increasingly clear that the mechanisms of disease are much more complex than we often understand, and relatively simple single-gene biomarkers for therapy targets are insufficient. Already, we are seeing progress in tackling these complex networks through multivalent antibodies and other advanced drug modalities. Harnessing the full benefit of these modalities will require cutting-edge technologies, such as AI/ML and DP/CP, including QCS CP, that can more efficiently distill intricate biology into actionable clinical decisions. Currently, 20% of oncology drugs in phase III development fall within this category; however, with nearly 40% of phase I therapies also fitting into this category, the need to shift to new diagnostic technologies is rapidly approaching (Figure 7).

Our findings illustrate a positive outlook for clinicians as advocates and future key stakeholders in the adoption of DP/CP and, more specifically, CP-based diagnostic tests. Enthusiasm for DP/CP is generally high despite varied degrees of awareness and low current utilization. There is potential for early consensus on the perceived benefits of DP/CP, as most clinicians agree on its potential to improve both key aspects of patient care and testing workflows. Finally, the majority of clinicians report being comfortable using both a CP-based test to interpret results independent of a pathologist, as well as using a CP-based CDx test for a novel biomarker.

However, despite the positive outlook, several contingencies must still be met for a new CP-based biomarker CDx test to be accepted in standard practice. The pathway to the adoption of a CP-based test has many parallels to traditional novel biomarker tests. Establishing KOL advocates and producing strong peer-reviewed clinical evidence are necessary to convince early-adopting clinicians; however, FDA approval and guideline inclusion are primarily viewed as sufficient for most clinicians to adopt. Further, for a small portion of clinicians, additional education on how the algorithms work may be required to address “black box” concerns.

Despite the enthusiasm observed in the survey results, securing the broad adoption of DP/CP-based tests will require coordinated efforts to alleviate clinician concerns from all additional stakeholders, including pathologists, biopharma, and regulatory bodies. Pathologists’ leading role in the adoption of DP/CP technologies means securing confidence with KOLs in their field is likely to impact oncologists’ willingness to order CP-based tests. This is supported by our findings as one of the leading influences for oncologists’ current use of CP-based tests. Creating and maintaining strong avenues for communication and collaboration between both parties will be essential for creating awareness of a new test and its implementation in the lab and clinical practice. Adoption will likely be a cyclic effort combining the review of performance data by pathologists, assessment of clinical impact by clinicians, and further review of aggregated results by all stakeholders. Biopharma researchers must collect and publicize data to enable clinicians and pathologists to independently evaluate test performance. Lastly, regulatory agencies can provide confidence to anyone concerned with “black box” concepts by establishing independent standards for evaluating and verifying CP-based tests, as well as providing independent approval.

While the evolution of these systems for traditional testing to better facilitate the adoption of DP/CP will be challenging, it is vital for the advancement of precision medicine. Without the adoption of these leading solutions, we risk remaining stagnant in the benefit we can provide to patients in need, limited to the constraints of simpler biomarkers and unable to develop insights from a more holistic perspective of the disease. We are already seeing the benefits of increasing digital pathology infrastructure through improved workflows, more consistent diagnoses, improved patient segmentation, and novel biomarker development. As such, it is clear that the broader adoption of both digital and computational pathology can become a key piece in ensuring that our diagnostic capabilities continue to evolve and usher in the next stage of precision medicine, further helping patients in need.

There are multiple potential next steps to encourage the adoption of DP/CP technologies. Clinicians and pathologists can collaborate to advocate for the necessary digital pathology infrastructure and protocol investments within their organizations, and subsequently for clinically meaningful computational pathology tests when available. The industry can align with clinicians and pathologists to raise awareness of technologies and identify the necessary specifications that will drive future adoption. Finally, industry and regulatory bodies can standardize the degree of evidence required for the approval of new technologies and methods, enabling regular improvements in the underlying AI algorithms to be produced and disseminated in a timely manner as the technology evolves.

There are inherent limitations to this study. As a self-reported qualitative survey, the results are reliant on the accuracy of all respondents’ answer selections. Further, the number of respondents may limit the generalizability of the results to the entire population of clinicians in the U.S. The sample size also restricts the ability to analyze statistical significance across stakeholder survey answers. Finally, this survey was exclusively conducted among medical oncologists, who may not have the same degree of technical knowledge of DP/CP as pathologists, and may also lack awareness of additional laboratory-specific considerations that influence the adoption of DP/CP-based tests. However, the intent of this survey was to specifically understand clinician perspectives on the technology and their willingness to adopt it as the intermediaries responsible for its utilization in patient care. Understanding their perspectives is critical for the holistic adoption of the technology. As such, technical questions were avoided, and questions were framed around the willingness to order tests. Previous work has focused specifically on the pathologist’s perspective [19]. Future work seeks to further explore both perspectives in parallel.

## 5. Conclusions

In conclusion, the findings of this study contribute to understanding of the clinician’s role in the adoption of DP/CP, particularly their perceptions of the value of CP-based tests and CP-based CDx tests. These results reflect the perspective of the stakeholders ultimately responsible for the ordering and uptake of any future tests. The future direction of this work is to continue monitoring the perceptions of CP-based tests among clinicians as they enter the clinic, thereby clarifying the benefits realized and the remaining barriers that still need to be addressed to maximize the impacts of digital and computational pathology.

## Figures and Tables

**Table 1:** Survey respondent demographics.

Cohort	Segment	Percentage of Respondents
All Clinicians	All	100%
Geography	Northeast	21%
	Midwest	22%
	South	36%
	West	22%
Practice Type	Academic Hospital	35%
	Community Hospital with Academic Affiliation	10%
	Community Hospital	20%
	Private Practice, Hospital, or Network-Affiliated	14%
	Independent Private Practice	22%
Years in Practice	0–1	6%
	2–10	36%
	11–20	30%
	21–40	29%
Anatomic Pathology Lab Most Commonly Used	In-House Lab	29%
	Affiliated Hospital Lab	5%
	Commercial Reference Lab	22%
	Specialty Reference Lab ^1^	45%

^1^ Specialty reference lab refers to labs conducting proprietary testing at a single site (e.g., Foundation Medicine and Natera), compared to commercial reference labs offering standard oncology testing services (e.g., Quest and LabCorp).

## Data Availability

The data underlying this article will be shared upon reasonable request to the corresponding author.

## Supplementary Materials

The following supporting information can be downloaded at: https://www.mdpi.com/article/10.3390/diagnostics15192527/s1, Figure S1: The utilization of current computational pathology testing; Figure S2: Strategies to alleviate concerns with DP/CP; Figure S3: Clinicians’ role in CP adoption decisions.

## Abbreviations

The following abbreviations are used in this manuscript:

DP/CP	Digital pathology and computational pathology
OOP	Out of pocket
CDx	Companion diagnostic
TROP2	Trophoblast cell surface antigen 2
