# Supplementary material for: Clinician Perspectives on Digital and Computational Pathology: Clinical Benefits, Concerns, and Willingness to Adopt
Source: Diagnostics (Basel). 2025 Oct 7;15(19):2527. doi: 10.3390/diagnostics15192527 (PMC12523993; doi:10.3390/diagnostics15192527)
Supplement: Supplementary file 1 [file diagnostics-15-02527-s001.zip › diagnostics-3836199-supplementary.PDF]

Clinician Perspectives on Digital and Computational Pathology: Clinical Benefits, Concerns, and Willingness to Adopt

Supplementary Tables and Figures

Supplementary Table 1. Key Queries and Survey Language

Key Query	Survey Question Language
Clinicians' current knowledge of availability and utilization of a computational pathology-based test	• Does the anatomic pathology lab you utilize offer any tests that use computational pathology today?<br>• Are you currently ordering diagnostic tests that employ computational pathology in any capacity for your patients?
Clinicians' current degree of comfort ordering a DP/CP-based test with increasing levels of automated interpretation and analysis	• What is your level of comfort using the results of a test for the following purposes if it were provided by either a digital or computational pathology technology, on a scale of 1 to 5, where 1 is not comfortable and 5 is very comfortable?
Clinicians' perception of the broad benefits of DP/CP	• To what degree do you agree or disagree that the adoption of digital and/or computational pathology could provide the following benefits to clinical practice, on a scale of 1 to 5, where 1 is strongly disagree and 5 is strongly agree?
Clinicians' perception of the potential barriers of a CP-based test	• (*Current Users)* At initial adoption of computational pathology-based diagnostic testing, what concerns did you have with receiving a test result from a computational pathology-based diagnostic, on a scale of 1 to 5, where 1 is not a concern, and 5 is high concern?<br>• *(Current non-Users)* Please rate your perceived influence the following barriers would have on adopting computational pathology solutions, on a scale of 1 to 5, where 1 is not influential and 5 is very influential.
Most impactful methods to alleviate concerns or barriers to ordering a CP-based test	• Please rate the level of impact each of the following measures could have on alleviating your hesitation to adopt computational pathology, on a scale of 1 to 5, where 1 is low impact, and 5 is high impact?
Clinicians' perceived role in adopting a CP-based test at their institution	• *(Current Users)* Were you part of the decision-making process to adopt

	computational pathology solutions at your organization?<br>• *(Current non-Users)* How involved would you be in the decision to adopt computational pathology at your institution?
Clinicians' willingness to order a theoretical CP-based CDx test, factors influencing decisions, and preference for a traditional CDx test vs. a CP-based CDx test	• How would you rate your level of comfort making a therapy decision for a lung cancer patient based on test results that use an algorithm like the one described to determine eligibility for a targeted therapy, on a scale of 1 to 5, where 1 is not comfortable and 5 is very comfortable?<br>• Please rate the impact of the following drivers on your ranking of being at least somewhat comfortable with using the described test, on a scale of 1 to 5, where 1 is a low impact, and 5 is a high impact<br>• If two companion diagnostic tests were available for the targeted therapy, a traditional pathology test and a computational pathology test, and the label did not specify which test to use, which test would you prefer to order?

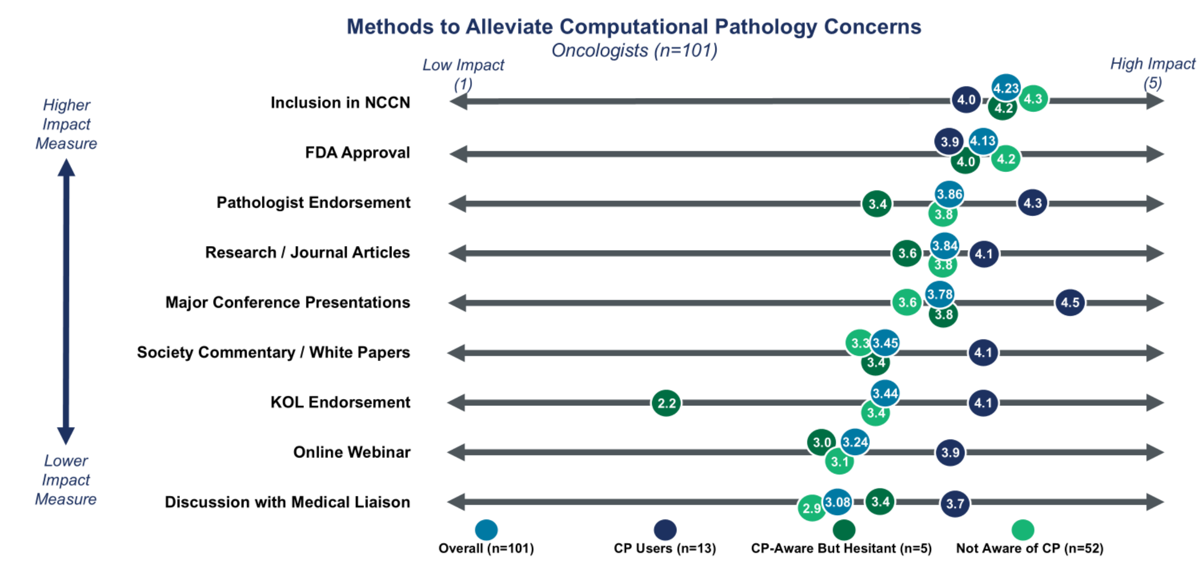

Supplementary Figure 2. Strategies to Alleviate Concerns with DP/CP. Regulatory approval is most likely to alleviate concerns around CP/DP use for clinicians. For current users, pathologist endorsement and dissemination of data through publications or presentations was most likely to address concerns.

Supplementary Figure 3.

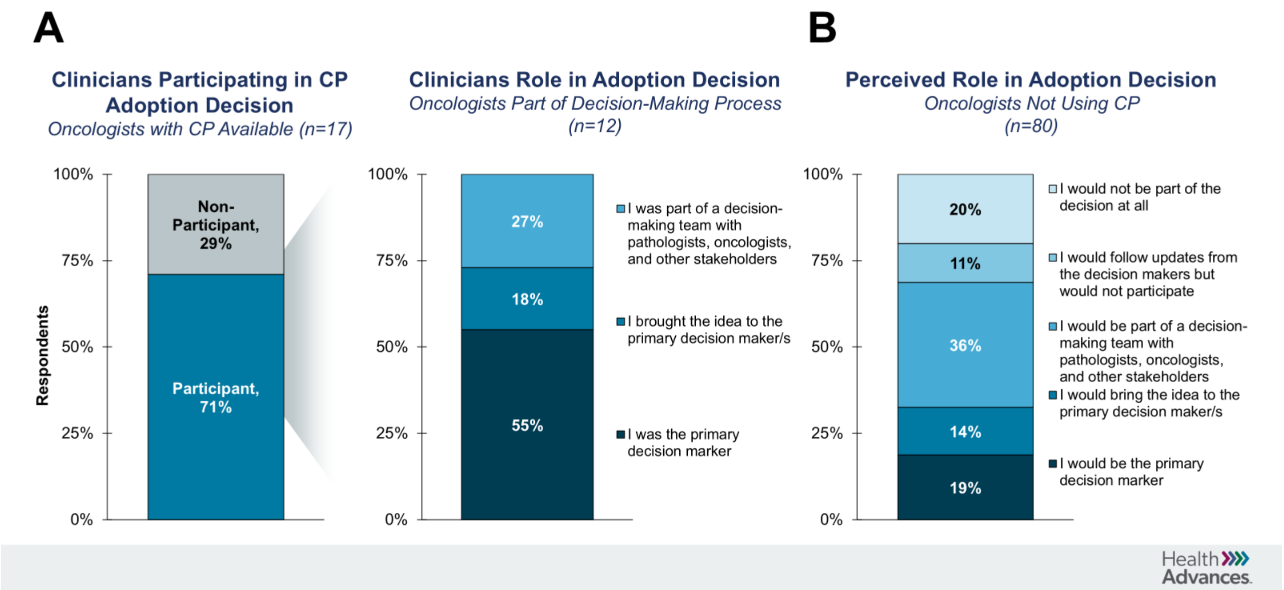

Supplementary Figure 3. Clinician Role in CP Adoption Decision. A) Clinicians that are users of CP/DP-based tests were likely to be a participant in the decision and perceive themselves as the primary decision maker, suggesting high engagement in adoption decisions. **B)** Clinicians not currently using CP also anticipate high engagement as the majority anticipate being actively involved in the decision process.
